# Heterodimeric Radiotracer Targeting PSMA and GRPR for Imaging of Prostate Cancer—Optimization of the Affinity towards PSMA by Linker Modification in Murine Model

**DOI:** 10.3390/pharmaceutics12070614

**Published:** 2020-07-01

**Authors:** Fanny Lundmark, Ayman Abouzayed, Bogdan Mitran, Sara S. Rinne, Zohreh Varasteh, Mats Larhed, Vladimir Tolmachev, Ulrika Rosenström, Anna Orlova

**Affiliations:** 1 Department of Medicinal Chemistry, Uppsala University, 751 23 Uppsala, Sweden; fanny.lundmark@ilk.uu.se (F.L.); ayman.abouzayed@ilk.uu.se (A.A.); bogdan.mitran@ki.se (B.M.); sara.rinne@ilk.uu.se (S.S.R.); zohreh.varasteh@tum.de (Z.V.); ulrika.rosenstrom@ilk.uu.se (U.R.); 2 Department of Clinical Neuroscience, Centre for Psychiatry Research, Karolinska Institutet and Stockholm County Council, 171 77 Stockholm, Sweden; 3 Department of Nuclear Medicine, Klinikum rechts der Isar der TUM, 80802 Munich, Germany; 4 Science for Life Laboratory, Department of Medicinal Chemistry, Uppsala University, 751 23 Uppsala, Sweden; mats.larhed@ilk.uu.se; 5 Department of Immunology, Genetics and Pathology, Uppsala University, 751 83 Uppsala, Sweden; vladimir.tolmachev@igp.uu.se; 6 Research Centrum for Oncotheranostics, Research School of Chemistry and Applied Biomedical Sciences, Tomsk Polytechnic University, 634050 Tomsk, Russia

**Keywords:** prostate cancer, PSMA, GRPR, heterodimer, molecular imaging, SPPS

## Abstract

Prostate-specific membrane antigen (PSMA) and gastrin-releasing peptide receptor (GRPR) are promising targets for molecular imaging of prostate cancer (PCa) lesions. Due to the heterogenic overexpression of PSMA and GRPR in PCa, a heterodimeric radiotracer with the ability to bind to both targets could be beneficial. Recently, our group reported the novel heterodimer BQ7800 consisting of a urea-based PSMA inhibitor, the peptide-based GRPR antagonist RM26 and NOTA chelator. The study reported herein, aimed to improve the affinity of BQ7800 towards PSMA by changing the composition of the two linkers connecting the PSMA- and GRPR-targeting motifs. Three novel heterodimeric analogues were synthesized by incorporation of phenylalanine in the functional linker of the PSMA-binding motif and/or shortening the PEG-linker coupled to RM26. The heterodimers were labeled with indium-111 and evaluated in vitro. In the competitive binding assay, BQ7812, featuring phenylalanine and shorter PEG-linker, demonstrated a nine-fold improved affinity towards PSMA. In the in vivo biodistribution study of [^111^In]In-BQ7812 in PC3-pip tumor-bearing mice (PSMA and GRPR positive), the activity uptake was two-fold higher in the tumor and three-fold higher in kidneys than for [^111^In]In-BQ7800. Herein, we showed that the affinity of a bispecific PSMA/GRPR heterodimer towards PSMA could be improved by linker modification.

## 1. Introduction

Prostate cancer (PCa) remains one of the most frequently diagnosed cancers worldwide, with almost 1.3 million new cases in 2018 [1]. It is a slowly progressive cancer form with both short- and long-term physical and emotional quality-of-life consequences [2]. Current methods used for diagnosis include measurement of prostate-specific antigen in blood, digital rectal examination and transrectal ultrasound-guided biopsy, which all have limited accuracy and can be painful for the patient. The methods are also limited in giving information regarding the spread of cancer. Since the choice of patient management is determined by the stage of PCa, accurate diagnosis is crucial for effective and successful treatment of the patient [3,4,5]. Therefore, new approaches such as molecular imaging using positron emission tomography (PET) or single photon emission computed tomography (SPECT) have gained a lot of focus since they are non-invasive and repetitive methods with high potential to improve diagnostic accuracy.

Several proteins have been identified as possible targets for molecular imaging of PCa lesions. Amongst these, prostate-specific membrane antigen (PSMA) has been highlighted as a promising specific target and received increased interest over the past years [6,7]. PSMA is a type II transmembrane glycosylated protein overexpressed on the surface of the majority of PCa cells. Enhanced expression of PSMA has been found in both poorly differentiated tumors as well as metastatic lesions and is usually increased with cancer progression [7]. Endogenous expression of PSMA can be found in proximal renal tubules, small intestine and salivary glands. However, the expression is significantly lower in healthy tissue than in PCa cells [8]. During the last years, numerous small-molecule inhibitors have shown promising results with high sensitivity and specificity [8,9,10]. Even though PSMA-targeting radioligands are promising, their use is limited to the expression of PSMA which has shown to vary in both primary tumors and distant metastases [11].

Another target overexpressed in PCa is gastrin-releasing peptide receptor (GRPR) [6,12]. It is a G-protein coupled receptor located in the cell membrane and is one of four members of the bombesin receptor family. Overexpression of GRPR has primarily been found in early stages of PCa and therefore, its enhanced expression is thought to be decreased with the progression of the disease. Endogenous expression of the receptor has been found in the pancreas, whereas only low levels have been detected in normal and hyperplastic prostate tissues [7]. Several GRPR-binding ligands have been developed during the past years, where antagonists have shown to be more beneficial as imaging agents than agonists due to higher tumor accumulation and absence of adverse effects, as well as no downregulation of GRPR or stimulation of tumor growth [13,14]. Clinical studies using GRPR-binding ligands have been able to visualize both primary tumors and lymph node metastases with high sensitivity [15,16,17].

Due to the complex biological process in PCa, it was suggested that no biological target alone would provide sufficient information for promoting the best health care for all patients suffering from PCa. Therefore, both PSMA and GRPR are highlighted as important targets to improve diagnostic accuracy, especially since their expression is considered very heterogeneous [18,19]. In a study investigating the distribution and heterogeneity of PSMA expression, a significant number of primary prostate carcinoma and distant metastases demonstrated high heterogeneity [11]. The heterogenic expression of these two targets was further confirmed in a small clinical study comparing [^68^Ga]Ga-RM2 and [^68^Ga]Ga-PSMA-11 in patients with biochemically recurrent PCa [19]. To address this, the use of a heterodimeric radiotracer targeting both PSMA and GRPR was proposed. In 2014, the first heterodimers combining the two pharmacophores by linking a urea-based PSMA inhibitor with a peptide-based GRPR agonist were reported [20,21]. Since then, three more heterodimers featuring the same molecular design have been developed, all showing promising results for imaging of PCa [22,23,24]. However, as mentioned above, using a GRPR-targeting antagonist has several advantages over an agonist. Based on this, our group recently reported two novel heterodimers containing a urea-based PSMA inhibitor and the peptide-based GRPR antagonist RM26; BO530 for labeling with radioiodine and NOTA-DUPA-RM26 (further denoted as BQ7800) for labeling with radiometals [25,26]. [^125^I]I-BO530, demonstrated high affinity to both GRPR and PSMA together with very long activity retention in tumors, especially for being labeled with a non-residualizing radiohalogen. Satisfactory tumor-to-organ ratios generated high contrast images in PSMA/GRPR-positive tumors in murine model up to 72 h pi. A relatively high initial kidney uptake was observed as a consequence of the high affinity towards PSMA [25]. [^111^In]In-BQ7800 demonstrated specific binding to both targets in vitro and in vivo, as well as relatively high tumor uptake, fast blood clearance and low uptake in normal tissue including kidneys. Compared with [^125^I]I-BO530, [^111^In]In-BQ7800 demonstrated high affinity towards GRPR but only modest affinity towards PSMA. Furthermore, it exhibited much shorter activity retention in tumor, potentially also reflecting the lower affinity towards PSMA.

**Figure 1:**
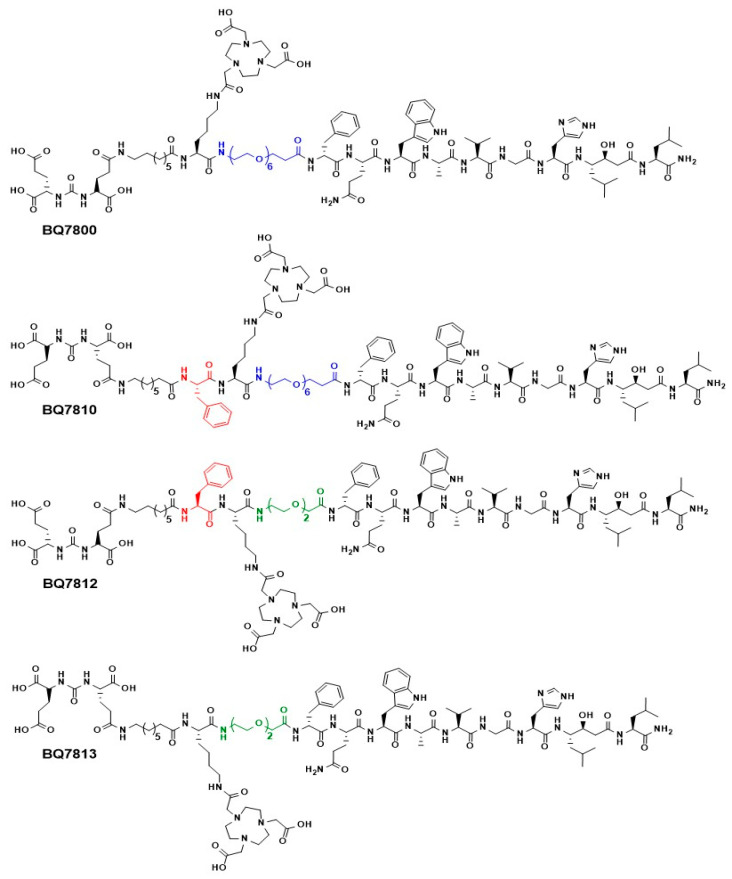
Chemical structure of the bispecific prostate-specific membrane antigen (PSMA)/gastrin-releasing peptide receptor (GRPR)-targeting heterodimers BQ7800, BQ7810, BQ7812 and BQ7813.

Despite promising results, further work focusing on the structure-activity relationships and kinetics of these relatively new bispecific radiotracers is needed. In the investigation reported herein, we synthesized three analogues based on BQ7800 (Figure 1) using the following two strategies (i) Since a hydrophobic functional linker is known to enhance the affinity towards PSMA, we decided to incorporate phenylalanine hypothesizing that its hydrophobic side chain will favorably interact with Phe546 and Trp541 in the entrance funnel to the active site [8,27]. (ii) The length of the PEG-linker has shown to not influence the affinity towards GRPR for monomers [28]. However, it has not been reported how it could affect the affinity towards either PSMA or GRPR for heterodimers. Therefore, alterations in the length of the PEG-linker coupled to the GRPR binding motif were explored to investigate how it influences the binding to both targets.

The overall aim of this study was to improve the affinity towards PSMA of the recently published heterodimer BQ7800 [26] and to investigate how the composition of both linkers influences the binding characteristics of the heterodimer.

## 2. Materials and Methods

Detailed information about the methods, instruments, solvents and chemicals can be found in the supporting information.

### 2.1. Synthesis

#### 2.1.1. (S)-5-(tert-butoxy)-4-(3-((S)-1,5-di-tert-butoxy-1,5-dioxopentan-2-yl)ureido)-5-oxopentanoic acid (R_2_-OH)

R_2_-OH was synthesized according to Figure S2, by dissolving triphosgene in dichloromethane (DCM) and sat. NaCO_3_ followed by addition of L-Glu(tBu)-O(tBu). The reaction was stirred vigorously for 30 min, whereafter the organic phase was separated from the aqueous phase, dried and evaporated. The crude was redissolved in DCM, followed by addition of L-Glu(OBn)-O(tBu) and Et_3_N. The reaction was left for 1 h. Solvent was evaporated and the crude was dissolved in EtOAc, washed with 2M KHSO_4_, dried, filtered and concentrated under reduced pressure. The crude was dissolved in EtOH followed by addition of 5% Pd/C (20% by mass) and the reaction was hydrogenated for 16 h. The mixture was filtered through a celite pad, concentrated under reduced pressure and used for the next step without further purification. Identity was confirmed by liquid chromatography-mass spectrometry (LC-MS); calculated [M + H]^1+^ 489.27, observed [M + H]^1+^ 489.2 (Figure S3) and nuclear magnetic resonance (NMR) spectroscopy; ^1^H NMR (400 MHz, Chloroform-*d*) δ 5.86 (d, 1H, NH), 5.43 (d, 1H, NH), 4.48–4.40 (m, 1H, CH), 4.36–4.29 (m, 1H, CH), 2.44–2.38 (m, 2H, CH_2_), 2.37–2.26 (m, 2H, CH_2_), 2.19–2.06 (m, 2H, CH_2_), 1.93–1.81 (m, 2H, CH_2_), 1.48 (s, 9H, CH_3_), 1.46 (s, 9H, CH_3_), 1.43 (s, 9H, CH_3_) (Figure S4). ^13^C NMR (101 MHz, Chloroform-*d*) δ 175.6, 172.7, 171.8, 158.1, 82.9, 82.4, 53.6, 53.3, 31.7, 30.8, 28.5, 28.4, 28.2, 28.2, 28.1 (Figure S5).

#### 2.1.2. On-Resin RM26

The synthesis of RM26 (D-Phe-Gln-Trp-Ala-Val-Gly-His-Sta-Leu-NH_2_) was carried out based on a previously published method [29], using Fmoc Rink Amide 4-Methylbenzhydrylamine (MBHA) resin (loading 0.69 mmol/g). After each coupling, the resin was washed with dimethylformamide (DMF), Fmoc removed by treatment of 20% piperidine in DMF and the resin was washed again with DMF. RM26 connected to the resin was used further to synthesize BQ7810, BQ7812 and BQ7813.

#### 2.1.3. BQ7810, BQ7812 and BQ7813

**Scheme 1:**
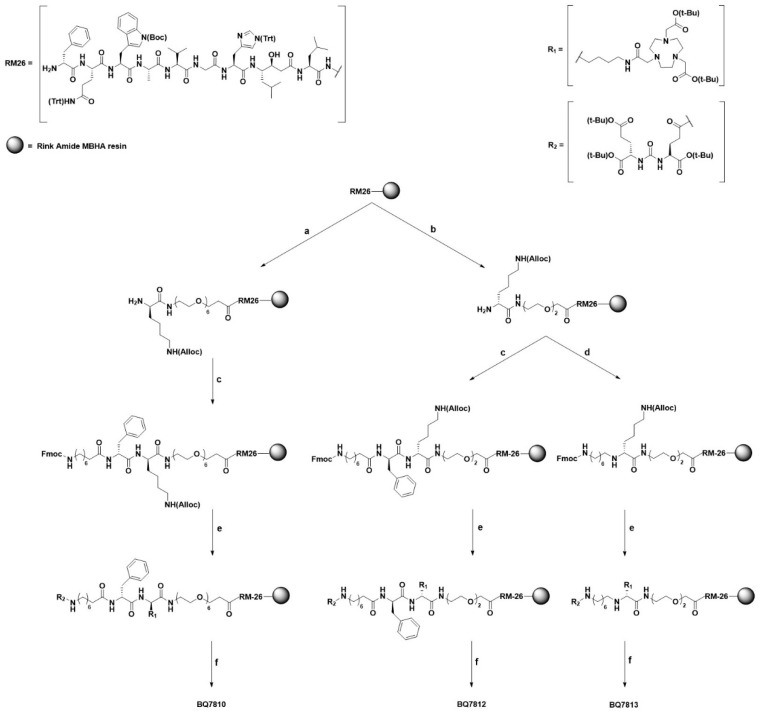
Solid-phase synthesis of BQ7810, BQ7812 and BQ7813 using Rink Amide 4-Methylbenzhydrylamine (MBHA) resin. (a) i. Fmoc-NH-PEG_6_-COOH ii. Fmoc-Lys(Alloc)-OH (b) i. Fmoc-O2Oc-OH ii. Fmoc-Lys(Alloc)-OH (c) i. Fmoc-Phe-OH ii. Fmoc-8-Aoc-OH (d) Fmoc-8-Aoc-OH (e) i. PhSiH_3_, Pd(PPh_3_)_4_, dichloromethane (DCM) ii. NOTA-bis(tBu)ester iii. R_2_-OH (f) trifluoroacetic acid (TFA), H_2_O, Et_3_SiH, thioanisole. Each coupling was performed in dimethylformamide (DMF) using benzotriazole-1-yl-oxy-tris-pyrrolidino-phosphonium hexafluorophosphate (PyBOP) and diisopropylethylamine (DIPEA). Fmoc was removed between each coupling using 20% piperidine in DMF.

Synthesis of BQ7810, BQ7812 and BQ7813 was performed on solid phase (Rink Amide MBHA resin) according to Scheme 1. All coupling reactions were performed in DMF using PyBOP and diisopropylethylamine (DIPEA). After each coupling, the resin was washed with DMF, Fmoc removed by treatment of 20% piperidine in DMF and the resin was washed again with DMF. First, Fmoc-NH-PEG_6_-COOH for BQ7810 and Fmoc-O2Oc-OH for BQ7812 and BQ7813, were coupled to resin-bound RM26. Thereafter, Fmoc-Lys(Alloc)-OH, Fmoc-Phe-OH and Fmoc-8-Aoc-OH were coupled one at the time for BQ7810 and BQ7812 and Fmoc-Lys(Alloc)-OH and Fmoc-8-Aoc-OH were coupled one at the time for BQ7813. Before the final Fmoc removal, Alloc side-chain protecting group on lysine was removed by addition of PhSiH_3_ and Pd(PPh_3_)_4_ in DCM. The reaction was left for 3 h and the resin was washed with DCM followed by coupling of NOTA-bis(tBu)ester using the same coupling condition as for the other couplings. Thereafter, Fmoc was removed and R_2_-OH was coupled as the final step. The products were cleaved from the resin, purified with preparative reversed-phase high performance liquid chromatography (RP-HPLC) and freeze-dried to yield 2.7 mg of BQ7810, 1.6 mg of BQ7812 and 0.93 mg of BQ7813. Identity and purity for all three compounds were confirmed with LC-MS—Calculated [M + 2H]^2+^ and [M + 3H]^3+^: 1227.2 and 818.4 for BQ7810, 1132.1 and 755.1 for BQ7812 (Figure S7), 1058.6 and 706.0 for BQ7813. Observed [M + 2H]^2+^ and [M + 3H]^3+^: 1227.2 and 818.4 for BQ7810 (Figure S6), 1132.0 and 754.9 for BQ7812 (Figure S7), 1058.4 and 705.9 for BQ7813 (Figure S8).

### 2.2. Radiolabeling

To an aqueous solution of the compound of interest (1 nmol), 0.2 M ammonium acetate buffer (pH 5.5) and [^111^In]InCl_3_ (25 MBq) were added and the reaction was left for 30 min at 85 °C. The molar activity was up to 25 MBq/nmol. The radiochemical yield was determined by instant thin-layer chromatography (ITLC) (0.2 M citric acid pH 2.0 as eluent). Labeling stability of each radiolabeled compound was evaluated in EDTA (1000-fold molar excess) and PBS and determined by ITLC at 1, 4 and 24 h incubation at room temperature.

### 2.3. Distribution Coefficient (LogD)

LogD was determined experimentally by addition of 10 pmol of the radiolabeled compound BQ7800, BQ7810, BQ7812 or BQ7813 to an Eppendorf tube containing n-octanol (500 µL) and water (500 µL). The tube was vortexed, centrifuged and fractions (100 µL) from each phase were collected and activity was measured using a gamma counter. The experiment was repeated twice for each compound.

### 2.4. In Vitro Experiments

Prostate carcinoma cell lines PC3 (GRPR positive) and LNCaP (PSMA positive) were obtained from American Type Culture Collection, ATCC via LGC Promochem, Borås, Sweden. Cells were cultured according to ATCC recommendations. The isogenic human prostate carcinoma cell line PC3-pip (GRPR and PSMA positive) was obtained from Dr. Warren Heston, Cleveland Clinic and was cultured as described earlier [25].

#### 2.4.1. In Vitro Specificity Test

In vitro binding specificity assay was performed using PC3 cells (GRPR+), LNCaP cells (PSMA+) and PC3-pip (GRPR+ and PSMA+) cells. Approximately 5 × 10^5^ cells/well were plated on six-well dishes 24 h prior to the experiment. To saturate the targets, cells were pre-incubated with non-labeled PSMA-11 (800 nM/well) and/or non-labeled NOTA-PEG_4_-RM26 (800 nM/well). For each compound, 20 nM/well of the radiolabeled heterodimer ([^111^In]In-BQ7810, [^111^In]In-BQ7812 or [^111^In]In-BQ7813) was added and the cells were incubated at 37 °C for 1 h. Thereafter, cells were detached and activity was measured using a gamma counter.

#### 2.4.2. In Vitro Cellular Processing

Cellular processing of BQ7810, BQ7812 and BQ7813 was performed on PC3-pip cells and analyzed at predetermined time points (1, 2, 4, 8 and 24 h). Cells were incubated with 20 nM/well of radiolabeled heterodimer ([^111^In]In-BQ7810, [^111^In]In-BQ7812 or [^111^In]In-BQ7813) at 37 °C. At each time point, the membrane-bound fractions were collected by addition of acidic wash solution (0.2 M glycine buffer, 0.15 M NaCl and 4 M urea). Thereafter, the internalized fractions were collected by the addition of a basic wash solution (1M NaOH). The activity in the collected fractions was measured using a gamma counter.

#### 2.4.3. In Vitro Competitive Binding Assay (IC_50_)

To compare the half-maximum inhibitory concentration (IC_50_) of BQ7800, BQ7810, BQ7812 and BQ7813, the experiments were run in parallel. The competitive binding assays were performed using PC3-pip cells. To block PSMA, cells were pre-incubated with PSMA-11 (300 nM/well) and to block GRPR, cells were pre-incubated with NOTA-PEG_4_-RM26 (300 nM/well). All four compounds were loaded with ^nat^In by the addition of InCl_3_ (30 min at 85 °C). To target GRPR, 1 nM of [^111^In]In-NOTA-PEG_4_-RM26 was added to each well followed by addition of an increased concentration (0-810 nM/well) of either ^nat^In-BQ7800, ^nat^In-BQ7810, ^nat^In-BQ7812 or ^nat^In-BQ7813. To target PSMA, 1 nM of [^111^In]In-PSMA-11 was added followed by addition of an increased concentration (0-8000 nM/well) of either ^nat^In-BQ7800, ^nat^In-BQ7810, ^nat^In-BQ7812, ^nat^In-BQ7813. Cells were incubated at 4 °C for 5 h. After incubation, cells were detached and activity content was measured using a gamma counter.

### 2.5. In Vivo Experiments

The in vivo experiments were performed using BALB/c nu/nu mice bearing PC3-pip xenografts. The mice were implanted with 5 × 10^6^ PC3-pip cells 12 days prior to the experiments. In vivo experiments were planned and performed in accordance with the national legislation on laboratory animals’ protection and were approved by the Ethics Committee for Animal Research in Uppsala, Sweden. PC3-pip tumor-bearing mice were injected with 40 pmol (30 kBq) of [^111^In]In-BQ7812 per mouse. Groups were also co-injected with 7.5 nmol of non-labeled NOTA-PEG_4_-RM26 and/or 7.5 nmol of non-labeled PSMA-11 to block GRPR and/or PSMA. Mice were euthanized at 1, 3 and 24 h pi. Organs of interest were collected, weighed and the activity uptake in each organ was measured using a gamma counter.

#### SPECT/CT Imaging

Whole-body SPECT/CT scans of mice bearing PC3-pip xenografts injected with [^111^In]In-BQ7812 (40 pmol, 830 kBq) only or in combination with 7.5 nmol of non-labeled NOTA-PEG_4_-RM26 and 7.5 nmol of non-labeled PSMA-11, were performed using nanoScan SPECT/CT (Mediso Medical Imaging Systems, Hungary). Imaging of the non-blocked group was performed at 1 and 3 h pi and for the GRPR/PSMA-blocked group at 1 h pi.

## 3. Results and Discussion

### 3.1. Synthesis and Radiolabeling

The bispecific PSMA/GRPR-targeting heterodimers BQ7810, BQ7812 and BQ7813 were successfully synthesized by manual solid-phase peptide synthesis (SPPS) and purified using RP-HPLC. Characterization with LC-MS observed [M + 2H]^2+^ and [M + 3H]^3+^ at *m/z* 1227.2 and 818.4 (BQ7810), 1132.0 and 754.9 (BQ7812) and 1058.4 and 705.9 (BQ7813), respectively. Synthesis and characterization of BQ7800 have already been reported [26]. The radiochemical yields after labeling with indium-111 were above 99% for all three new analogues and only minimal amount (<1.3 ± 0.4%) of free indium-111 could be detected after incubation with a high excess of EDTA up to 24 h post labeling. This indicates high stability of the [^111^In][In-NOTA]-complex which is desirable for an imaging agent. Low labeling stability could result in an elevated activity uptake in blood, lungs and bones, which in return would decrease the imaging contrast.

### 3.2. In Vitro Characterization

**Figure 2:**
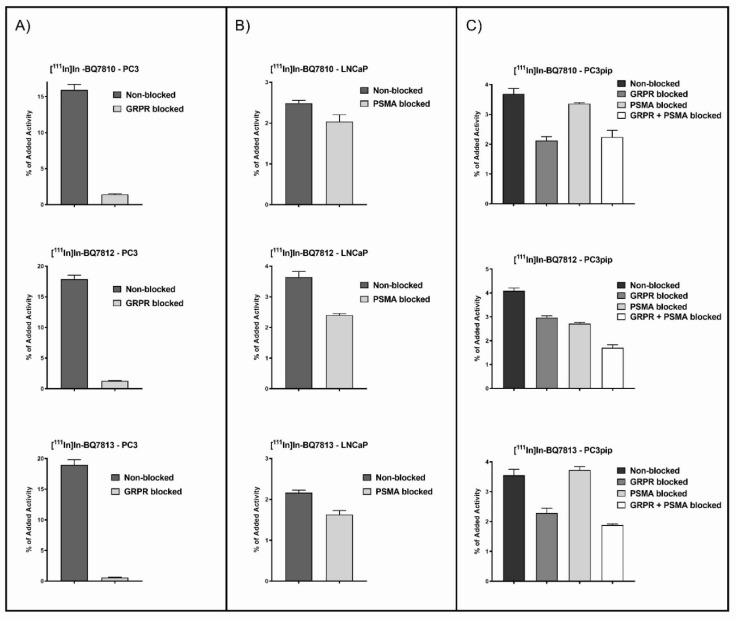
In vitro binding specificity of indium-111 labeled BQ7810, BQ7812 and BQ7813 in (**A**) PC3 cells (GRPR positive), (**B**) LNCaP cells (PSMA positive), (**C**) PC3-pip cells (GRPR and PSMA positive). Non-labeled NOTA-PEG_4_-RM26 (800 nM/well) was used to block GRPR (**A**,**C**) and non-labeled PSMA-11 (800 nM/well) was used to block PSMA (**B**,**C**).

To investigate binding specificity of [^111^In]In-BQ7810, [^111^In]In-BQ7812 and [^111^In]In-BQ7813 to PSMA and GRPR, an in vitro specificity test was performed using PC3 (GRPR+), LNCaP (PSMA+) and PC3-pip (GRPR+ and PSMA+) cells (Figure 2). Activity uptake in PC3 cells pre-incubated with an excess of non-labeled NOTA-PEG_4_-RM26 was significantly reduced compared with the non-treated cells for all compounds, indicating specific binding to GRPR. Specific binding to PSMA could also be observed for [^111^In]In-BQ7812 and [^111^In]In-BQ7813 in LNCaP cells pre-incubated with non-labeled PSMA-11, even though the effect was less pronounced than for GRPR. The same tendency was observed for [^111^In]In-BQ7810, although the decrease in activity uptake in pre-treated LNCaP cells was not statistically significant (*p* = 0.062). When using PC3-pip cells, which express both targets, the uptake of [^111^In]In-BQ7812 and [^111^In]In-BQ7813 was significantly lower when both targets were pre-blocked than when only one target was blocked. This could not be seen for [^111^In]In-BQ7810, where blocking of both targets showed a similar effect as blocking GRPR alone. The less pronounced blocking effect for PSMA for all analogues could probably be explained by the lower affinity towards PSMA than towards GRPR. This tendency was also reported for BQ7800 [26].

Since all three heterodimers consist of a PSMA inhibitor connected to a GRPR antagonist, the cell internalization was expected to be rather slow. Results from the cellular processing study showed rapid binding to the targets on the cell surface for all three compounds. Approximately 20% of the cell-associated activity was internalized after 24 h incubation (Figure S1). The results were in good agreement with the results reported for BQ7800 [26] and indicate that the composition of the linkers does not affect the cellular processing of the heterodimers significantly.

A competitive binding assay was performed to investigate if a more hydrophobic functional linker could improve the affinity towards PSMA and how the length of the PEG-linker affects the binding to both targets (Table 1). All four compounds had similar IC_50_ values in the low nanomolar range towards GRPR, consistent with the published data for the corresponding GRPR-targeting monomers [28]. Thus, the length of the PEG-linker, ranging from PEG_2_ to PEG_6_, does not influence the binding towards GRPR to a large extent for either monomers or heterodimers. Regarding the IC_50_ values towards PSMA, only BQ7812 had a lower value than BQ7800. An explanation hereto could be the structural modification in the functional linker of BQ7812. The incorporation of phenylalanine seems to increase the hydrophobic interactions with Phe546 and Trp541 in the entrance funnel to the active site of PSMA. In contrast to the unaffected affinity towards GRPR, the PEG-length surprisingly affected the binding to PSMA, taking into account its position in the heterodimer (Figure 1). BQ7810 and BQ7812 consist of the same PSMA binding motif containing phenylalanine in the same position but differ in the length of the PEG-linker, containing PEG_6_ and PEG_2_ respectively. As seen in Table 1, BQ7812 demonstrated a higher affinity towards PSMA compared with BQ7810, resulting in a 10-fold better IC_50_ value. A longer PEG-linker allows for more flexibility in the molecule and thereby more steric hindrance which could negatively affect the binding to PSMA. Since both BQ7810 and BQ7813 had IC_50_ values >1 µM, only BQ7812 was taken further for evaluation and comparison with BQ7800 in vivo.

### 3.3. In Vivo Characterization

**Figure 3:**
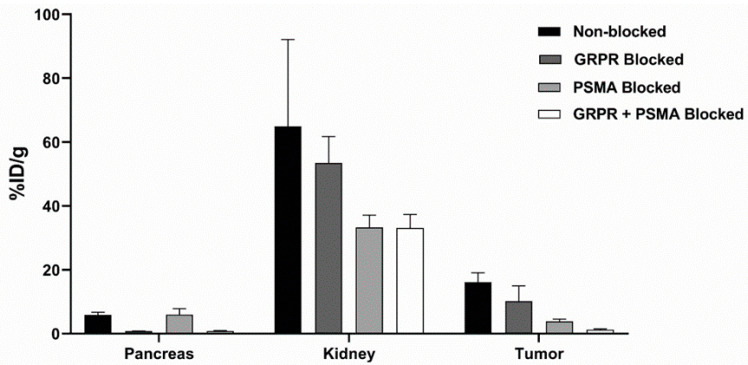
In vivo binding specificity of indium-111 labeled BQ7812 towards PSMA and GRPR at 1 h pi. in PC3-pip tumor-bearing mice. Co-injection of non-labeled NOTA-PEG_4_-RM26 (7.5 nmol/animal) was used to block GRPR and co-injection of non-labeled PSMA-11 (7.5 nmol/animal) was used to block PSMA.

Specific binding of [^111^In]In-BQ7812 to PSMA and GRPR was demonstrated in the in vivo specificity test on PC3-pip tumor-bearing mice (Figure 3). Activity uptake in the tumors was significantly decreased in the group co-injected with 7.5 nmol of non-labeled PSMA-11 compared with the non-blocked group, thus demonstrating specific binding to PSMA. In the kidneys, a strong tendency to decreased activity uptake was observed for the PSMA-blocked group. This was expected due to the endogenous expression of PSMA in this organ. However, it must be noted that the difference is not statistically significant due to high data spread, which could be explained by interindividual variability in kidney uptake shortly after injection (1 h pi.). Regarding the specificity to GRPR, a significant decrease in activity uptake was seen in the pancreas in the group co-injected with 7.5 nmol of non-labeled NOTA-PEG_4_-RM26 compared with the non-blocked group. Since the pancreas has an endogenous expression of GRPR, specific binding was demonstrated. When comparing the activity uptake in tumors between the non-blocked and the GRPR blocked group, the decrease was not statistically significant (*p* = 0.192). A possible reason therefore could be the higher expression of PSMA than GRPR in the PC3-pip tumor cells. However, a significant decrease in uptake of activity could be seen in the group co-injected with both non-labeled PSMA-11 and NOTA-PEG_4_-RM26 compared with the group co-injected with only PSMA-11, confirming specific binding of [^111^In]In-BQ7812 towards GRPR.

**Figure 4:**
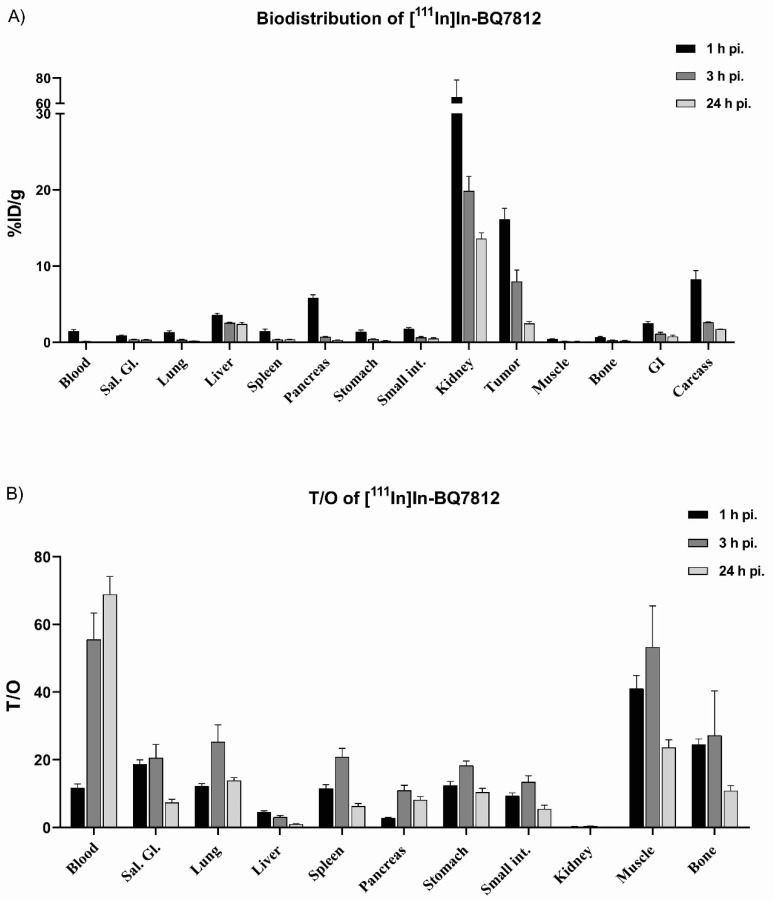
(**A**) Biodistribution profile of indium-111 labeled BQ7812 at 1, 3 and 24 h pi. in PC3-pip tumor-bearing mice. (**B**) tumor-to-organ ratios of indium-111 labeled BQ7812 at 1, 3 and 24 h pi.

Results from the biodistribution study over time in PC3-pip tumor-bearing mice are shown in Figure 4 (details in Tables S1 and S2). At 1 h pi., an elevated uptake of [^111^In]In-BQ7812 was found in the kidneys, pancreas, liver and tumor, with the highest uptake in kidneys (64.9%ID/g) followed by the tumor (16.1%ID/g). Uptake of [^111^In]In-BQ7812 rapidly decreased over time and already at 3 h pi., almost no activity was found in studied organs except in the liver, kidneys and tumor (Figure 4A). Thus, the activity uptake decreased with time in the tumor as well as in all studied organs and tissues except the liver. Tumor-to-organ ratios (T/O) were the highest at 3 h pi. since the activity was rapidly eliminated from blood (Figure 4B). High tumor-to-organ ratios are important for imaging contrast and accurate diagnosis and staging of PCa. High tumor-to-blood ratio determines the overall contrast whereas high tumor-to-intestine, -muscle and –bone ratios simplify the image interpretation in the anatomical context of PCa. As seen in Figure 4A,B, uptake in normal organs was low and tumor-to-organ ratios were high 3 h pi., making it a suitable time point for imaging.

**Figure 5:**
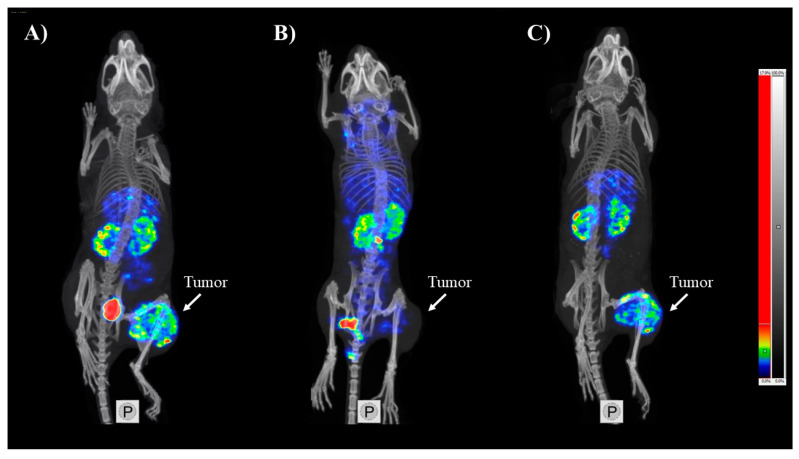
NanoScan SPECT/CT images of PC3-pip tumor-bearing mice after injection of [^111^In]In-BQ7812. (**A**) Non-blocked 1 h pi. (**B**) Blocked with co-injection of non-labeled PSMA-11 and NOTA-PEG_4_-RM26, 1 h pi. (**C**) Non-blocked 3 h pi.

Imaging of PC3-pip tumor-bearing mice using nanoScan SPECT/CT was performed at 1 and 3 h pi. and the generated images were in good agreement with the ex vivo data (Figure 5). Due to the high uptake of [^111^In]In-BQ7812, the tumor could be visualized already at 1 h pi. and the only healthy organs with high activity uptake at this time point were the kidneys. Activity cleared from healthy organs and blood with time, leading to an improved imaging contrast at 3 h pi. Co-injection of non-labeled PSMA-11 and NOTA-PEG_4_-RM26 resulted in a decreased kidney uptake and a negligible activity uptake in the tumor (Figure 5B). This, together with the results from the in vitro and in vivo specificity tests, confirmed the specific binding of [^111^In]In-BQ7812 to both PSMA and GRPR.

### 3.4. Comparison of [^111^In]In-BQ7800 and [^111^In]In-BQ7812 Heterodimers

**Figure 6:**
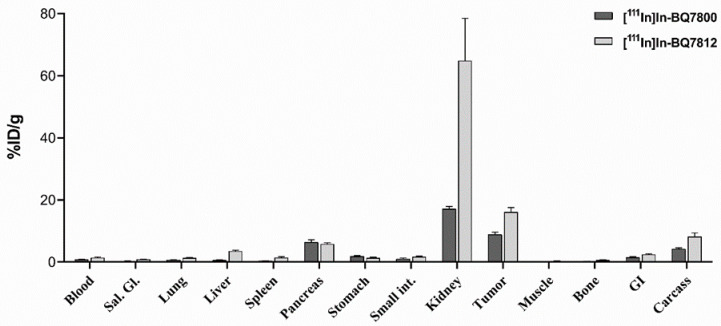
Biodistribution profile of indium-111 labeled BQ7800 [26] and BQ7812 at 1 h pi. in PC3-pip tumor-bearing mice.

The difference in the molecular design of the heterodimers BQ7800 and BQ7812 is the composition of the linkers connecting the PSMA and GRPR binding motifs (Figure 1). Compared with BQ7800, BQ7812 consists of a shorter PEG_2_- instead of a PEG_6_-linker connected to the GRPR binding motif, as well as phenylalanine in the functional linker of the PSMA binding part. Both heterodimers were labeled with high radiochemical yields, formed stable radionuclide/chelator-complexes and demonstrated specific binding to both targets in vitro and in vivo. In the competitive binding assay (Table 1), no pronounced difference in affinity towards GRPR was observed for these two heterodimers. However, when investigating the binding towards PSMA, the IC_50_ value of BQ7812 was almost one order of magnitude better than BQ7800, thus indicating an enhanced affinity due to the incorporation of phenylalanine. Furthermore, it was observed that not only the structure of the functional linker seems to influence the binding to PSMA but also the composition of the PEG-linker. As described above, a reason for this could be an increase in steric hindrance due to the longer and more flexible molecule. Another explanation for the difference in the binding capacity could be that changes in the PEG-linker also affect the overall hydrophobicity of the molecule. BQ7812 is more hydrophobic than BQ7800 (Table 1), which seems to benefit the binding to PSMA. A comparison of the biodistribution profiles of [^111^In]In-BQ7800 and [^111^In]In-BQ7812 1 h pi. showed that the uptake of [^111^In]In-BQ7812 was two-fold higher in the tumor and three-fold higher in kidneys which corroborates with the higher affinity towards PSMA (Figure 6). The activity uptake in liver was also significantly higher than that for [^111^In]In-BQ7800 which may be caused by the more hydrophobic nature of BQ7812 (Figure 1 and Table 1). However, despite the lower expression of PSMA in healthy tissue, the increase of activity uptake in tumors was lower than in the kidneys. This could be explained by the difference in availability of PSMA in the tumor and the kidneys, where tissue penetration probably is not required to the same extent in the kidneys as in the tumor.

When designing the novel PSMA/GRPR-targeting heterodimers investigated in this study, we hypothesized that an increased affinity towards PSMA would result in longer tumor retention, since the first heterodimer reported by our group ([^125^I]I-BO530) demonstrated high affinity to PSMA combined with very good retention in tumor [25]. Unfortunately, no enhanced tumor retention could be seen for [^111^In]In-BQ7812 compared to [^111^In]In-BQ7800. The rapid washout of [^111^In]In-BQ7812 contradicts the previous findings for [^125^I]I-BO530, which had comparable affinities to both targets (100 ± 10 nM towards PSMA and 20 ± 2 nM towards GRPR). However, these molecules were labeled with different radionuclides, which also could have an impact on both the overall activity uptake and the retention in tumors. BQ7812 contains the hydrophilic [^111^In][In-NOTA]-complex, while BO530 contains the hydrophobic radioiodinated tyrosine. Taken together, the behavior of PSMA/GRPR-targeting heterodimers is complexed since both the hydrophobicity in the functional linker as well as of the whole molecule, the length of the PEG-linker and the choice of radionuclide seem to have an impact on both biodistribution and in vivo targeting.

Comparison of the biodistribution pattern of the newly designed heterodimer with available numerical data for [^68^Ga]Ga-Glu-urea-Lys(Ahx)-HBED-CC-BZH_3_ [20] and [^68^Ga]Ga-Glu-urea-(His-Glu)_2_-HBED-CC-PEG_2_-BZH_3_ [22]_,_ showed that [^111^In]In-BQ7812 had similar rapid clearance via the renal pathway. Renal uptake of [^111^In]In-BQ7812 was similar to the hydrophilized heterodimer [^68^Ga]Ga-Glu-urea-(His-Glu)_2_-HBED-CC-PEG_2_-BZH_3_ but approximately two-fold lower than the non-hydrophilized heterodimer. In agreement with the antagonistic nature of the GRPR-binding motif of BQ7812, its uptake in pancreas was lower than for heterodimers based on agonists. The hepatic uptake of [^111^In]In-BQ7812 was also higher, however, tumor-to-liver ratio was over five and activity excretion to the gastrointestinal tract was minimal, which would allow clear visualization of lesions in the low abdomen. For comparison, activity uptake of [^64^Cu]Cu-[DUPA-6-Ahx-(NODAGA)-5-Ava-BBN(7–14)NH_2_] in the liver, kidneys and intestine at 18 h pi was much higher than for [^111^In]In-BQ7812 at 1 h pi [21]. Unfortunately, it is not possible to compare tumor activity uptake of [^111^In]In-BQ7812 with other heterodimers tested separately in LNCaP (PSMA positive) and PC-3 (GRPR positive) xenografts but as already mentioned, activity uptake of [^111^In]In-BQ7812 in tumor was two-fold higher than that for [^111^In]In-BQ7800, the parental heterodimer with lower affinity to PSMA.

## 4. Conclusions

Herein, we have shown that modifications in the molecular design can be used to improve the characteristics of a heterodimer targeting PSMA and GRPR. The length of the PEG-linker connected to the GRPR binding motif of the heterodimer was shown to affect the binding to PSMA but not to GRPR that, together with the incorporation of hydrophobic phenylalanine in the functional linker of the PSMA binding motif, led to a 10-fold improved affinity towards PSMA and high activity uptake in tumors. However, further investigations are needed to develop an optimal PSMA/GRPR-targeting heterodimer with high affinity to both targets, together with a preferable pharmacokinetic profile with high uptake and long retention in the tumor, low uptake in normal tissue and fast blood clearance.

## Figures and Tables

**Table 1:** IC_50_ values for BQ7800, BQ7810, BQ7812 and BQ7813 and logD for indium-111 labeled BQ7800, BQ7810, BQ7812 and BQ7813. 1 nM of [^111^In]In-NOTA-PEG_4_-RM26 or [^111^In]In-PSMA-11 were used as displacement radioligands in the competitive binding assay.

Parameter	BQ7800	BQ7810	BQ7812	BQ7813
IC_50_ (GRPR)	4 ± 1 nM	3 ± 1 nM	6 ± 3 nM	8 ± 2 nM
IC_50_ (PSMA)	921 ± 363 nM	>1000 nM	102 ± 80 nM	>1000 nM
LogD	−2.62	−1.47	−1.44	−2.27

## Supplementary Materials

The following are available online at https://www.mdpi.com/1999-4923/12/7/614/s1, Figure S1: Cellular processing of [^111^In]In-BQ7810, [^111^In]In-BQ7812, and [^111^In]In-BQ7813 using PC3-pip cells. Figure S2: Synthesis of (S)-5-(tert-butoxy)-4-(3-((S)-1,5-di-tert-butoxy-1,5-dioxopentan-2-yl)ureido)-5-oxopentanoic acid (R_2_-OH). Figure S3: MS of (S)-5-(tert-butoxy)-4-(3-((S)-1,5-di-tert-butoxy-1,5-dioxopentan-2-yl)ureido)-5-oxopentanoic acid (R_2_-OH). Figure S4: ^1^H NMR spectrum of (S)-5-(tert-butoxy)-4-(3-((S)-1,5-di-tert-butoxy-1,5-dioxopentan-2-yl)ureido)-5-oxopentanoic acid (R_2_-OH). Figure S5: ^13^C NMR spectrum of (S)-5-(tert-butoxy)-4-(3-((S)-1,5-di-tert-butoxy-1,5-dioxopentan-2-yl)ureido)-5-oxopentanoic acid (R_2_-OH).Figure S6: A) Analytical HPLC (UV detection at 214 and 254 nm) of BQ7810. B) MS of BQ7810. Figure S7: A) Analytical HPLC (UV detection at 214 and 254 nm) of BQ7812. B) MS of BQ7812. Figure S8: A) Analytical HPLC (UV detection at 214 and 254 nm) of BQ7813. B) MS of BQ7813. Table S1: In vivo biodistribution of [^111^In]In-BQ7812 (40 pmol/animal, 30 kBq) in BALB/c nu/nu mice bearing PC3-pip xenografts at 1, 3, and 24 h pi. Activity uptake was calculated as percent injected dose per tissue weight (%ID/g) and data are presented as average ± standard deviation. Table S2: Tumor-to-organ ratios of [^111^In]In-BQ7812 (40pmol/animal, 30 kBq) in BALB/c nu/nu mice bearing PC3-pip xenografts at 1, 3, and 24 h pi. Data are presented as average ± standard deviation.

## References

[1] Bray F., Ferlay J., Soerjomataram I., Siegel R.L., Torre L.A., Jemal A. (2018). Global cancer statistics 2018: GLOBOCAN estimates of incidence and mortality worldwide for 36 cancers in 185 countries. CA Cancer J. Clin..

[2] Saad F. (2019). Quality of life in men with prostate cancer. Lancet Oncol..

[3] Attard G., Parker C., Eeles R.A., Schröder F., Tomlins S.A., Tannock I., Drake C.G., De Bono J.S. (2016). Prostate cancer. Lancet.

[4] Lavery A., Kirby R.S., Chowdhury S. (2016). Prostate cancer. Medicine (Baltimore).

[5] Mottet N., Bellmunt J., Bolla M., Briers E., Cumberbatch M.G., De Santis M., Fossati N., Gross T., Henry A.M., Joniau S. (2017). EAU-ESTRO-SIOG Guidelines on Prostate Cancer. Part 1: Screening, Diagnosis, and Local Treatment with Curative Intent. Eur. Urol..

[6] Rybalov M., Ananias H.J., Hoving H.D., Van der Poel H.G., Rosati S., de Jong I.J. (2014). PSMA, EpCAM, VEGF and GRPR as imaging targets in locally recurrent prostate cancer after radiotherapy. Int. J. Mol. Sci..

[7] Barve A., Jin W., Cheng K. (2014). Prostate cancer relevant antigens and enzymes for targeted drug delivery. J. Control. Release.

[8] Kiess A.P., Banerjee S.R., Mease R.C., Rowe S.P., Rao A., Foss C.A., Chen Y., Yang X., Cho S.Y., Nimmagadda S. (2015). Prostate-specific membrane antigen as a target for cancer imaging and therapy. Q. J. Nucl. Med. Mol. Imaging.

[9] Mease R.C., Foss C.A., Pomper M.G. (2014). PET Imaging in Prostate Cancer: Focus on Prostate-Specific Membrane Antigen. Curr. Top. Med. Chem..

[10] Donin N.M., Reiter R.E. (2017). Why Targeting PSMA Is a Game Changer in the Management of Prostate Cancer. J. Nucl. Med..

[11] Mannweiler S., Amersdorfer P., Trajanoski S., Terrett J.A., King D., Mehes G. (2009). Heterogeneity of prostate-specific membrane antigen (PSMA) expression in prostate carcinoma with distant metastasis. Pathol. Oncol. Res..

[12] Baratto L., Jadvar H., Iagaru A. (2017). Prostate Cancer Theranostics Targeting Gastrin-Releasing Peptide Receptors. Mol. Imaging Biol..

[13] Cheng S., Lang L., Wang Z., Jacobson O., Yung B.C., Zhu G., Gu D., Ma Y., Zhu X., Niu G. (2017). PET Imaging of Prostate Cancer with Ga-68 Labeled GRPR Agonist BBN7-14 and Antagonist RM26. Bioconjugate Chem..

[14] Millar J.B., Rozengurt E. (1990). Chronic desensitization to bombesin by progressive down-regulation of bombesin receptors in Swiss 3T3 cells. Distinction from acute desensitization. J. Biol. Chem..

[15] Kahkonen E., Jambor I., Kemppainen J., Lehtio K., Gronroos T.J., Kuisma A., Luoto P., Sipila H.J., Tolvanen T., Alanen K. (2013). In vivo imaging of prostate cancer using [68Ga]-labeled bombesin analog BAY86-7548. Clin. Cancer Res..

[16] Minamimoto R., Sonni I., Hancock S., Vasanawala S., Loening A., Gambhir S.S., Iagaru A. (2018). Prospective evaluation of 68 Ga-RM2 PET/MRI in patients with biochemical recurrence of prostate cancer and negative findings on conventional imaging. J. Nucl. Med..

[17] Nock B.A., Kaloudi A., Lymperis E., Giarika A., Kulkarni H.R., Klette I., Singh A., Krenning E.P., De Jong M., Maina T. (2017). Theranostic perspectives in prostate cancer with the gastrin-releasing peptide receptor antagonist NeoBOMB1: Preclinical and first clinical results. J. Nucl. Med..

[18] Iagaru A. (2017). Will GRPR Compete with PSMA as a Target in Prostate Cancer?. J. Nucl. Med..

[19] Minamimoto R., Hancock S., Schneider B., Chin F.T., Jamali M., Loening A., Vasanawala S., Gambhir S.S., Iagaru A. (2016). Pilot comparison of 68Ga-RM2 PET and 68Ga-PSMA-11 PET in patients with biochemically recurrent prostate cancer. J. Nucl. Med..

[20] Eder M., Schäfer M., Bauder-Wüst U., Haberkorn U., Eisenhut M., Kopka K. (2014). Preclinical evaluation of a bispecific low-molecular heterodimer targeting both PSMA and GRPR for improved PET imaging and therapy of prostate cancer. Prostate.

[21] Bandari R.P., Jiang Z., Reynolds T.S., Bernskoetter N.E., Szczodroski A.F., Bassuner K.J., Kirkpatrick D.L., Rold T.L., Sieckman G.L., Hoffman T.J. (2014). Synthesis and biological evaluation of copper-64 radiolabeled [DUPA-6-Ahx-(NODAGA)-5-Ava-BBN(7-14)NH2], a novel bivalent targeting vector having affinity for two distinct biomarkers (GRPr/PSMA) of prostate cancer. Nucl. Med. Biol..

[22] Liolios C., Schäfer M., Haberkorn U., Eder M., Kopka K. (2016). Novel Bispecific PSMA/GRPr Targeting Radioligands with Optimized Pharmacokinetics for Improved PET Imaging of Prostate Cancer. Bioconjugate Chem..

[23] Escudero-Castellanos A., Ocampo-García B., Morales-Ávila E., Luna-Gutiérrez M., Isaac-Olivé K., Ferro-Flores G., Santos-Cuevas C. (2018). Synthesis and preclinical evaluation of the 177Lu-DOTA-PSMA(inhibitor)-Lys3-bombesin heterodimer designed as a radiotheranostic probe for prostate cancer. Nucl. Med. Commun..

[24] Mendoza-Figueroa M.J., Escudero-Castellanos A., Ramirez-Nava G.J., Ocampo-García B.E., Santos-Cuevas C.L., Ferro-Flores G., Pedraza-Lopez M., Avila-Rodriguez M.A. (2018). Preparation and preclinical evaluation of 68Ga-iPSMA-BN as a potential heterodimeric radiotracer for PET-imaging of prostate cancer. J. Radioanal. Nucl. Chem..

[25] Abouzayed A., Yim C.-B., Mitran B., Rinne S.S., Tolmachev V., Larhed M., Rosenström U., Orlova A. (2019). Synthesis and Preclinical Evaluation of Radio-Iodinated GRPR/PSMA Bispecific Heterodimers for the Theranostics Application in Prostate Cancer. Pharmaceutics.

[26] Mitran B., Varasteh Z., Abouzayed A., Rinne S.S., Puuvuori E., De Rosa M., Larhed M., Tolmachev V., Orlova A., Rosenström U. (2019). Bispecific GRPR-Antagonistic Anti-PSMA/GRPR Heterodimer for PET and SPECT Diagnostic Imaging of Prostate Cancer. Cancers.

[27] Kopka K., Benešová M., Bařinka C., Haberkorn U., Babich J. (2017). Glu-ureido-based inhibitors of prostate-specific membrane antigen: Lessons learned during the development of a novel class of low-molecular-weight theranostic radiotracers. J. Nucl. Med..

[28] Varasteh Z., Rosenström U., Velikyan I., Mitran B., Altai M., Honarvar H., Rosestedt M., Lindeberg G., Sörensen J., Larhed M. (2014). The effect of mini-PEG-based spacer length on binding and pharmacokinetic properties of a 68Ga-labeled NOTA-conjugated antagonistic analog of bombesin. Molecules.

[29] Varasteh Z., Velikyan I., Lindeberg G., Sörensen J., Larhed M., Sandström M., Selvaraju R.K., Malmberg J., Tolmachev V., Orlova A. (2013). Synthesis and characterization of a high-affinity NOTA-conjugated bombesin antagonist for GRPR-targeted tumor imaging. Bioconjugate Chem..

