# Supplementary material for: Heterodimeric Radiotracer Targeting PSMA and GRPR for Imaging of Prostate Cancer—Optimization of the Affinity towards PSMA by Linker Modification in Murine Model
Source: Pharmaceutics. 2020 Jul 1;12(7):614. doi: 10.3390/pharmaceutics12070614 (PMC7408065; doi:10.3390/pharmaceutics12070614)
Supplement: Supplementary file 1 [file pharmaceutics-12-00614-s001.pdf]

pharmaceutics

Supplementary Materials: Heterodimeric Radiotracer Targeting PSMA and GRPR for Imaging of Prostate Cancer—Optimization of the Affinity towards PSMA by Linker Modification in Murine Model

Fanny Lundmark [1,†], Ayman Abouzayed [1,†], Bogdan Mitran [1,2], Sara S. Rinne [1], Zohreh Varasteh [1,3], Mats Larhed [4], Vladimir Tolmachev [5,6], Ulrika Rosenström [1,‡] and Anna Orlova [1,4,6,*,‡]

1 Department of Medicinal Chemistry, Uppsala University, 751 23 Uppsala, Sweden; fanny.lundmark@ilk.uu.se (F.L.); ayman.abouzayed@ilk.uu.se (A.A.); bogdan.mitran@ki.se (B.M.); sara.rinne@ilk.uu.se (S.S.R.); zohreh.varasteh@tum.de (Z.V.); ulrika.rosenstrom@ilk.uu.se (U.R.)
2 Department of Clinical Neuroscience, Centre for Psychiatry Research, Karolinska Institutet and Stockholm County Council, SE-171 77 Stockholm, Sweden
3 Department of Nuclear Medicine, Klinikum rechts der Isar der TUM, 80802 Munich, Germany
4 Science for Life Laboratory, Department of Medicinal Chemistry, Uppsala University, 751 23 Uppsala, Sweden; mats.larhed@ilk.uu.se
5 Department of Immunology, Genetics and Pathology, Uppsala University, 751 83 Uppsala, Sweden; vladimir.tolmachev@igp.uu.se
6 Research Centrum for Oncotheranostics, Research School of Chemistry and Applied Biomedical Sciences, Tomsk Polythechnic University, 634050 Tomsk, Russia
* Correspondence: anna.orlova@ilk.uu.se; Tel.: +46(0)18-4715303
† These authors contributed equally
‡ These authors contributed equally

Received: 8 June 2020; Accepted: 28 June 2020; Published: date

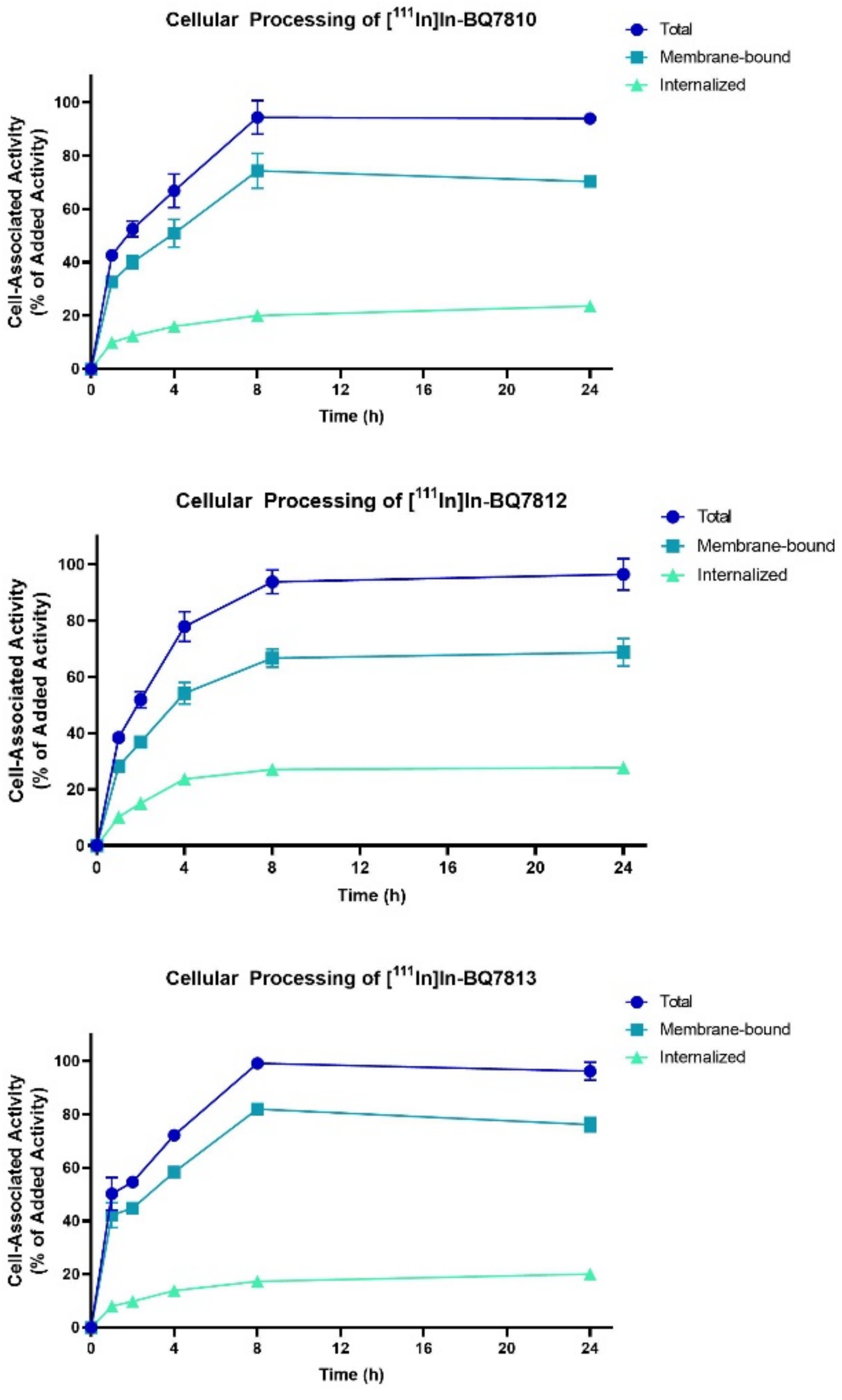

Figure S1. Cellular processing of [111In]In-BQ7810, [111In]In-BQ7812, and [111In]In-BQ7813 using PC3-pip cells.

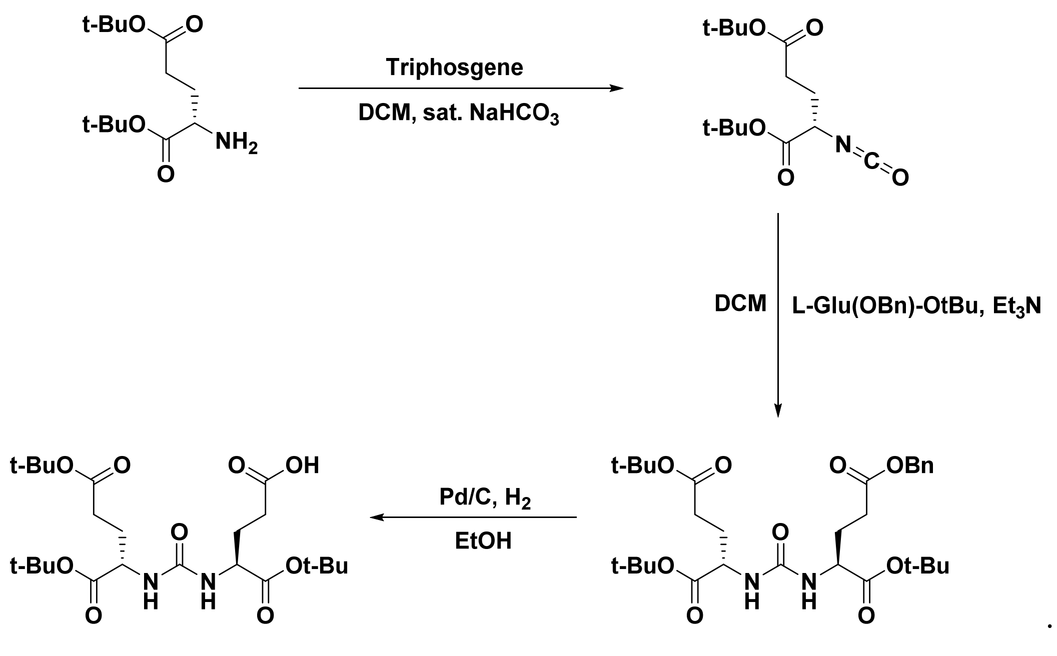

Figure S2. Synthesis of (S)-5-(tert-butoxy)-4-(3-((S)-1,5-di-tert-butoxy-1,5-dioxopentan-2-yl)ureido)-5-oxopentanoic acid (R_2-OH).

Table S1. In vivo biodistribution of [^{111}In]In-BQ7812 (40 pmol/animal, 30 kBq) in BALB/c nu/nu mice bearing PC3-pip xenografts at 1, 3, and 24 h pi. Activity uptake was calculated as percent injected dose per tissue weight (%ID/g) and data are presented as average ± standard deviation.

Organ	[^{111}In]In-BQ7812		
	**1 h**	**3 h**	**24 h**
Blood	1. 44 ± 0.47	0.14 ± 0.02	0.04 ± 0.01
Salivary glands	0.88 ± 0.20	0.39 ± 0.08	0.34 ± 0.05
Lung	1.34 ± 0,36	0.34 ± 0.15	0.18 ± 0.03
Liver	3.56 ± 0.55	2.54 ± 0.22	2.40 ± 0.44
Spleen	1.48 ± 0.51	0.38 ± 0.09	0.40 ± 0.05
Pancreas	5.83 ± 0,83	0.71 ± 0.08	0.30 ± 0.03
Stomach	1.36 ± 0.53	0.43 ± 0.12	0.24 ± 0.03
Small intestine	1.76 ± 0.35	0.64 ± 0.27	0.51 ± 0.22
Kidney	64.87 ± 27.26	19.86 ±3.80	13.60 ± 1.54
Tumor	16.10 ± 2.96	7.96 ± 3.04	2.48 ± 0.48
Muscle	0.40 ± 0.10	0.15 ± 0.02	0.11 ± 0.03
Bone	0.67 ± 0.19	0.27 ± 0.08	0.22 ± 0.06
GI	2.51 ± 0.42	1.13 ± 0.39	0.77 ± 0.35
Carcass	8.24 ± 2.37	2.61 ± 0.16	1.74 ± 0.03

Table S2. Tumor-to-organ ratios of [^{111}In]In-BQ7812 (40pmol/animal, 30 kBq) in BALB/c nu/nu mice bearing PC3-pip xenografts at 1, 3, and 24 h pi. Data are presented as average ± standard deviation.

Organ	[^{111}In]In-BQ7812		
	**1 h**	**3 h**	**24 h**
Blood	11.69 ± 2.30	55.52 ± 15.62	68.91 ± 10.45
Salivary glands	18.68 ± 2.60	20.56 ± 7.98	7.39 ± 1.88
Lung	12.28 ± 1.34	25.30 ± 10.02	13.91 ± 1.65
Liver	4.56 ± 0.77	3.08 ± 1.00	1.04 ± 0.15
Spleen	11.49 ± 2.37	20.84 ± 5.10	6.32 ± 1.59
Pancreas	2.77 ± 0.43	10.94 ± 3.05	8.19 ± 1.92
Stomach	12.46 ± 2.36	18.28 ± 2.80	10.46 ± 2.28
Small intestine	9.31 ± 1.80	13.42 ± 3.68	5.52 ± 2.17
Kidney	0.27 ± 0.07	0.39 ± 0.09	0.18 ± 0.02
Muscle	41.04 ± 7.76	53.27 ± 24.41	23.65 ± 4.58
Bone	24.52 ± 3.25	35.14 ± 25.72	10.93 ± 2.46

General Information

Instruments and Equipment

Analytical high performance liquid chromatography (HPLC) was performed on a Dionex UltiMate 3000 HPLC system with a Bruker amazon SL ion trap mass spectrometer and detection by UV (diode array detector, 214, 254, and 280 nm) and electrospray ionization (ESI) MS using a Penomenex Kinetex C18 column (50 × 3.0 mm, 2.6 µm particle size, 100 Å pore size) with gradients of H_2O/CH_3CN/0.05%HCOOH as mobile phase at a flow rate of 1.5 mL/min. Preparative reversed-phase high-performance liquid chromatography (RP-HPLC) was performed by UV-trigged (254 nm) fraction collection with a Glison HPLC system using a Machery-nagel NUCLEODUR C18 HTec column (21 × 125 mm, particle size 5 µm) and H_2O/CH_3CN/0.1%TFA as mobile phase at a flow rate of 10 mL/min. Nuclear magnetic resonance (NMR) spectra were recorded at 25 °C at 400 MHz for ^{1}H and at 101 MHz for ^{13}C. Chemical shifts are reported in ppm with the residual solvent peak as internal standard ($CDCl_3$; ^{1}H 7.26 ppm, ^{13}C 77.16 ppm). nanoScan SPECT/CT were performed using indium-111 energy window, 256 × 256 matrix, and 20 min acquisition time; CT scans were acquired at the

following parameters: 50 keV, 670 μA, 480 projections, 5 min acquisition time. SPECT raw data were reconstructed using Tera-Tomo™ 3D SPECT. CT raw data were reconstructed using Nucline 2.03 Software (Mediso Medical Imaging Systems, Hungary). Activity content was measured using an automated gamma counter (3-inch NaI(Tl) detector, 2480 Wizard2, PerkinElmer). Statistical analysis were performed by unpaired, two-tailed t-test using GraphPad Prism 8 for Windows (GraphPad Software Inc, San Diego, CA, USA), p values < 0.05 were considered statistical significant.

Chemicals and Solvents

All starting materials and solvents were purchased from Sigma Aldrich, Fisher Scientific, and Honeywell, and used without further purification if nothing else stated. NOTAbis(tBu)ester was purchased from CheMatech, France; Fmoc Rink Amide MBHA resin (loading 0.69 mmol/g), L-Glu(OBn)-O(tBu), and Fmoc-NH-PEG$_6$-COOH were purchased from Iris Biotech Gmbh (Marktredwitz, Germany); PyBOP was purchased from Novabiochem, Switzerland.

Characterization

(S)-5-(tert-butoxy)-4-(3-((S)-1,5-di-tert-butoxy-1,5-dioxopentan-2-yl)ureido)-5-oxopentanoic acid (R_2-OH)

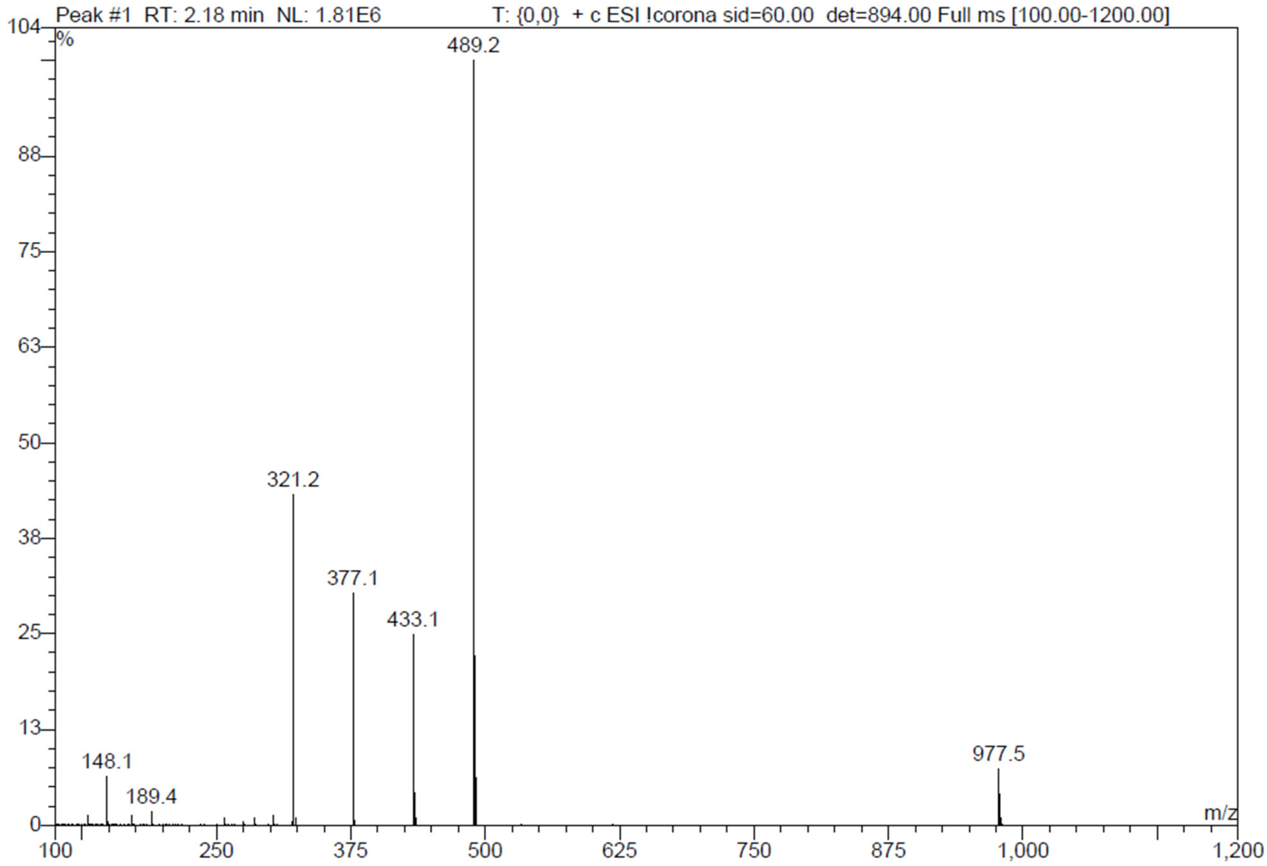

Figure S3. MS of (S)-5-(tert-butoxy)-4-(3-((S)-1,5-di-tert-butoxy-1,5-dioxopentan-2-yl)ureido)-5-oxopentanoic acid (R_2-OH).

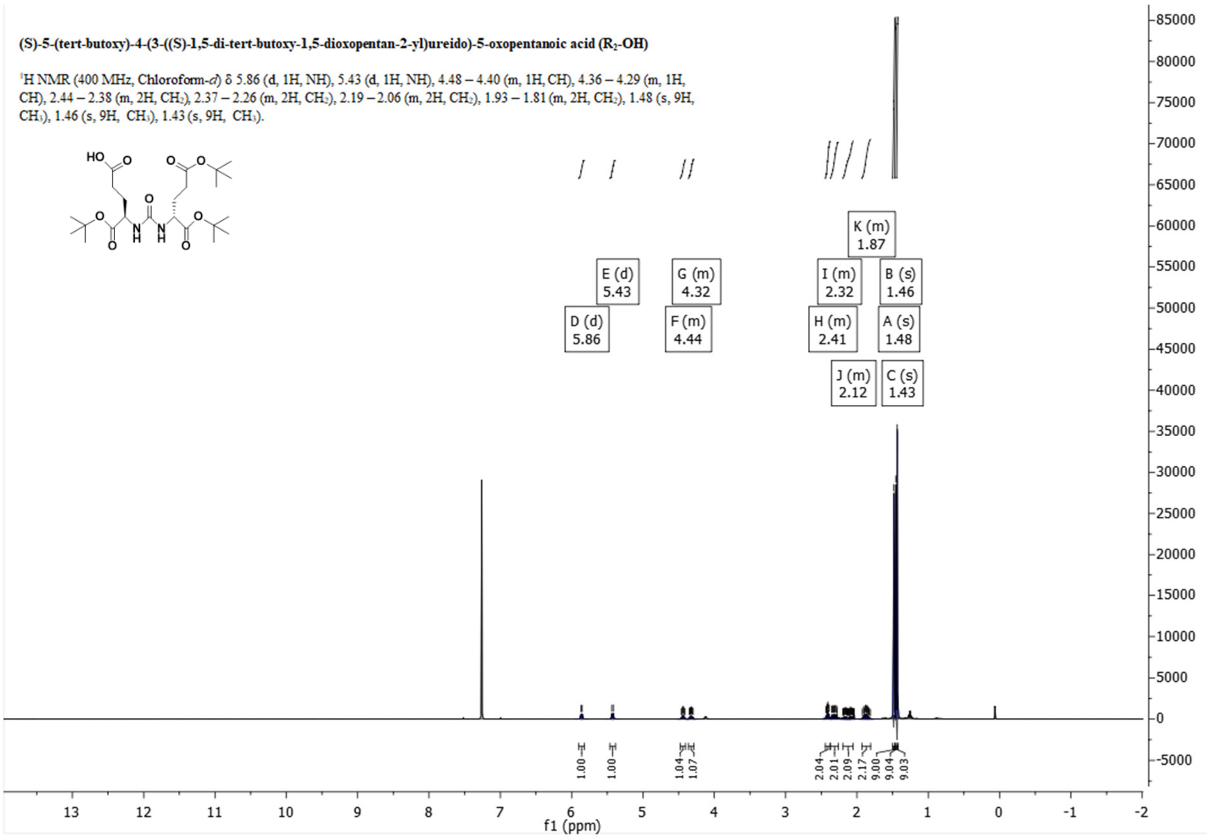

Figure S4. ^{1}H NMR spectrum of (S)-5-(tert-butoxy)-4-(3-((S)-1,5-di-tert-butoxy-1,5-dioxopentan-2-yl)ureido)-5-oxopentanoic acid (R_2-OH).

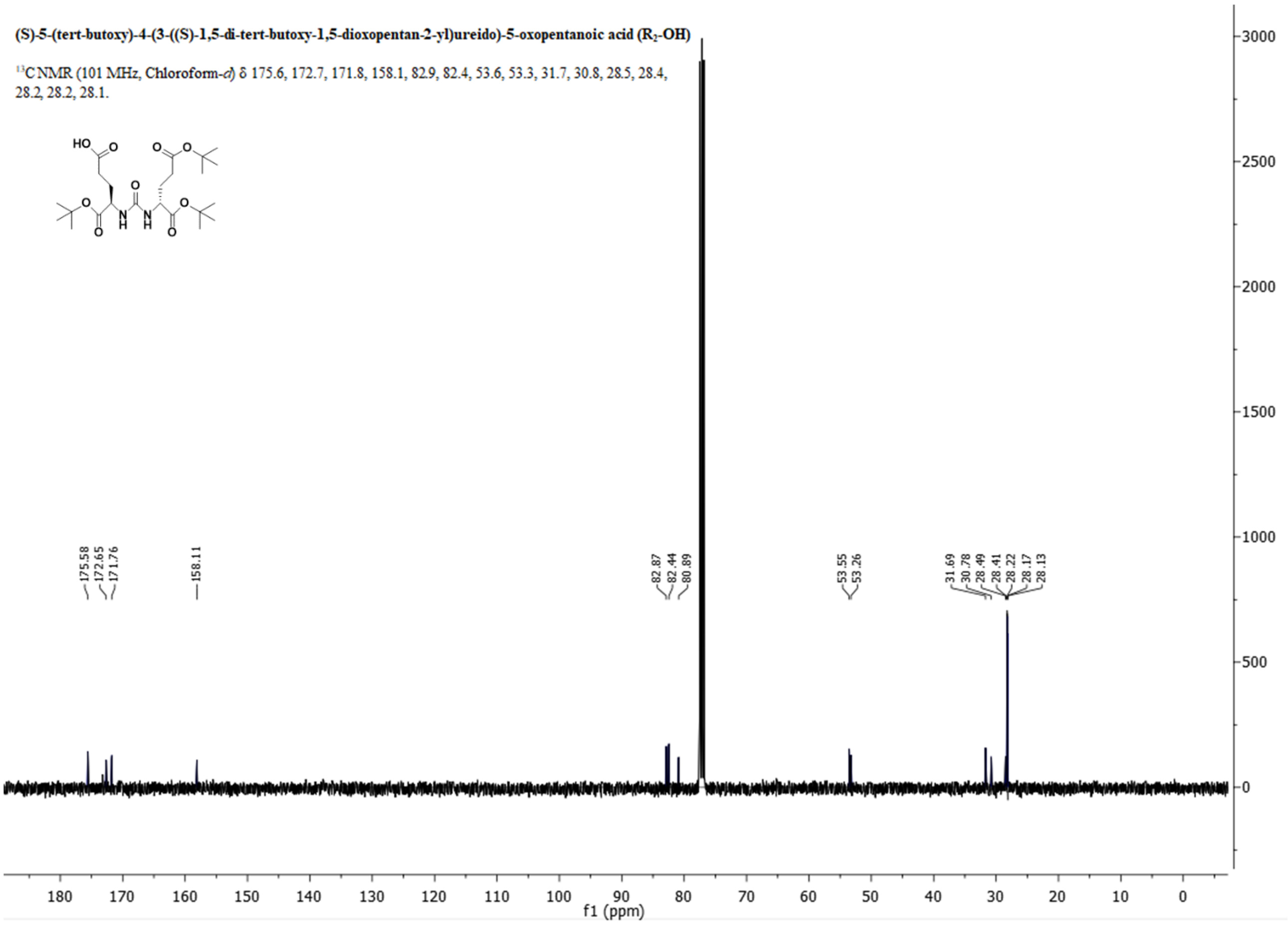

Figure S5. ^{13}C NMR spectrum of (S)-5-(tert-butoxy)-4-(3-((S)-1,5-di-tert-butoxy-1,5-dioxopentan-2-yl)ureido)-5-oxopentanoic acid (R_2-OH).

BQ7810

A)

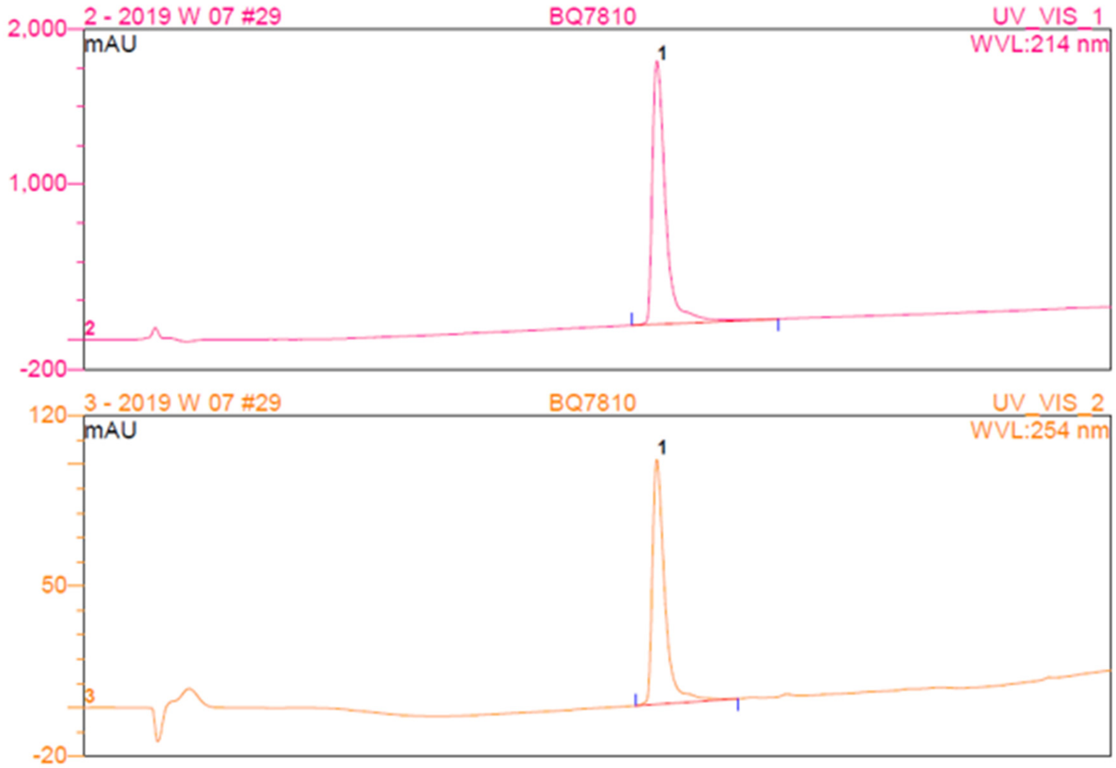

B)

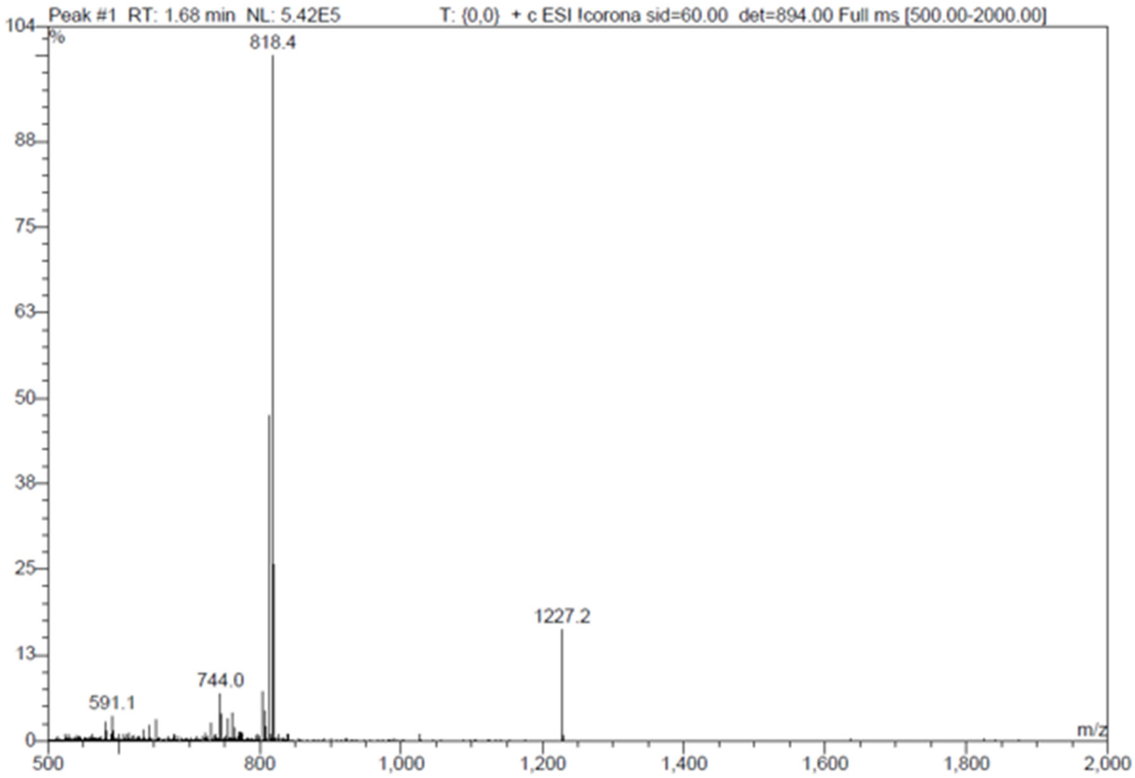

Figure S6. A) Analytical HPLC (UV detection at 214 and 254 nm) of BQ7810. B) MS of BQ7810.

BQ7812

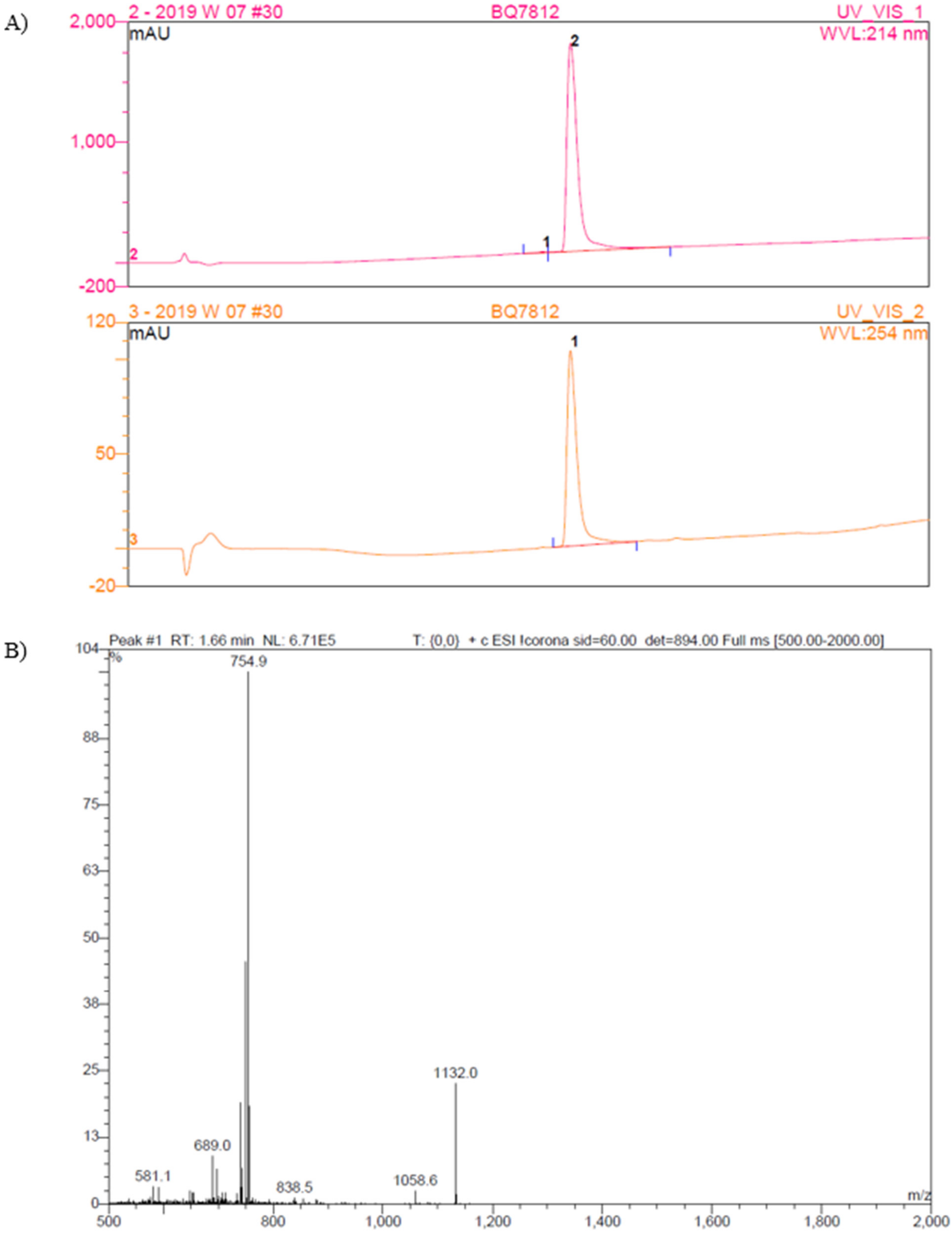

Figure S7. A) Analytical HPLC (UV detection at 214 and 254 nm) of BQ7812. B) MS of BQ7812.

BQ7813

A)

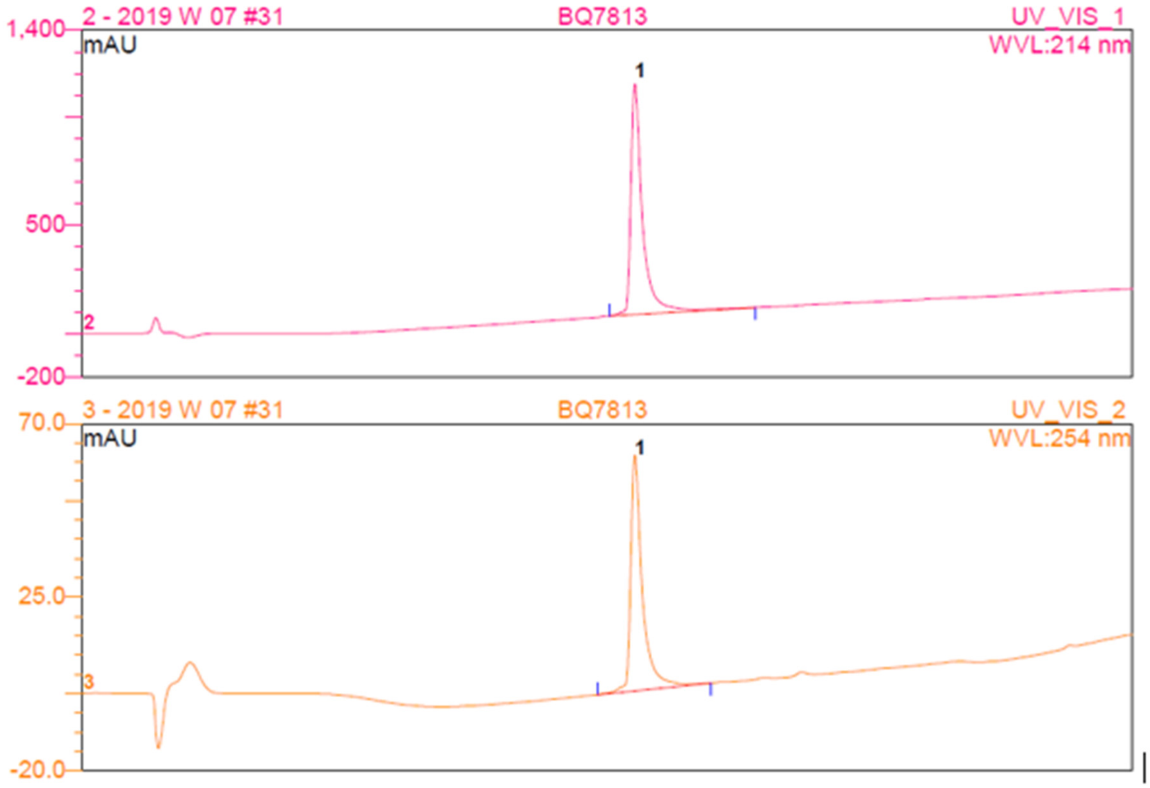

B)

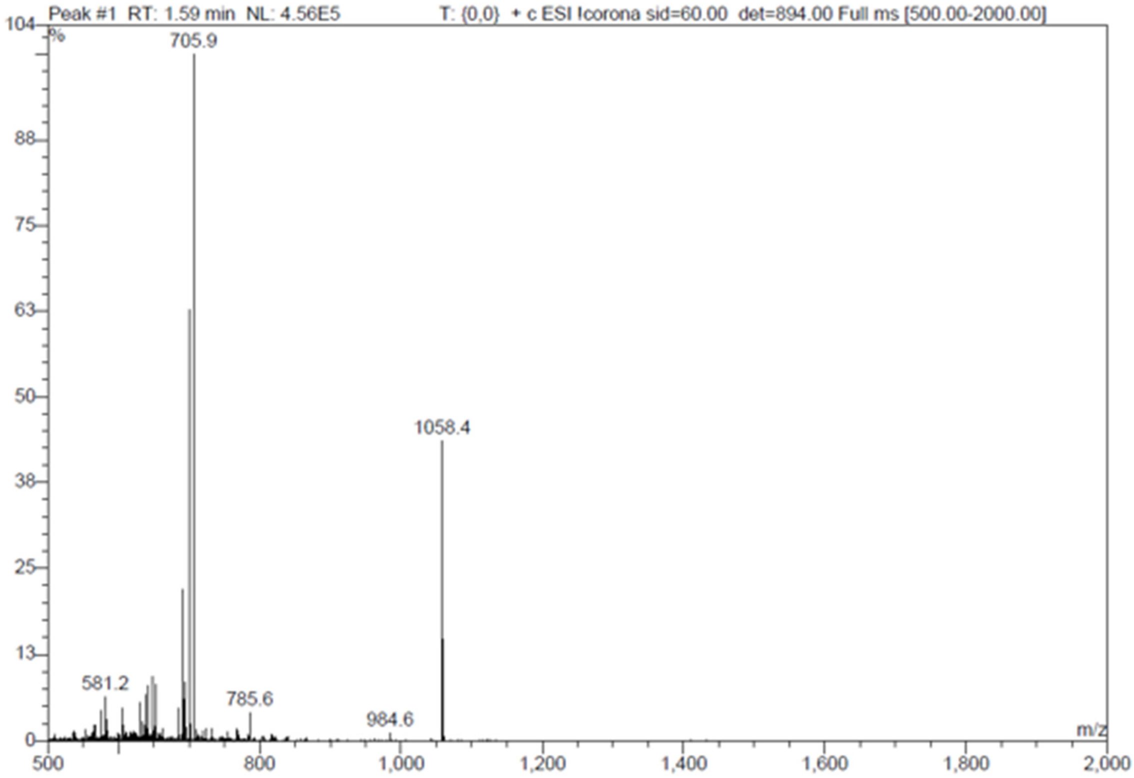

Figure S8. A) Analytical HPLC (UV detection at 214 and 254 nm) of BQ7813. B) MS of BQ7813.
